# Soluble interleukin-27 receptor alpha is a valuable prognostic biomarker for acute graft-versus-host disease after allogeneic haematopoietic stem cell transplantation

**DOI:** 10.1038/s41598-018-28614-4

**Published:** 2018-07-09

**Authors:** Shuangzhu Liu, Jingjing Han, Huanle Gong, Yongsheng Li, Xiebing Bao, Jiaqian Qi, Hong Liu, Jia Chen, Xiaojin Wu, Yang Xu, Shoubao Ma, Depei Wu

**Affiliations:** 1 Jiangsu Institute of Hematology, The First Affiliated Hospital of Soochow University, Suzhou, 215006 China; 2 Institute of Blood and Marrow Transplantation, Soochow University, Suzhou, 215123 China; 3 Collaborative Innovation Center of Hematology, Soochow University, Suzhou, 215006 China; 4 Department of Rheumatology, Huai’an First People’s Hospital, Nanjing Medical University, Huai’an, 223300 China

## Abstract

Acute graft-versus-host disease (aGVHD) is a major life-threatening complication after allogeneic haematopoietic stem cell transplantation. Interleukin-27 receptor alpha (IL-27Rα) is a co-receptor of IL-27, an inflammatory cytokine that possesses extensive immunological functions. It has been reported that IL-27Rα can exist in its soluble form (sIL-27Rα) in human serum and can function as a natural IL-27 antagonist. In this study, we examined serum sIL-27Rα levels and evaluated their prognostic value in aGVHD. A total of 152 subjects were prospectively recruited and separated into the training group (n = 72) and the validation group (n = 80). Serum sIL-27Rα at neutrophil engraftment was measured by ELISA. In the training set, a cut-off value of sIL-27Rα = 59.40 ng/ml was identified to predict grade II–IV aGVHD (AUC = 0.735, 95% CI 0.618–0.853, *P* = 0.001). Cumulative incidences of grade II–IV aGVHD (*P* = 0.004), relapse rate (*P* = 0.008), and non-relapse mortality (*P* = 0.008) in patients with low serum sIL-27Rα (≥59.40 ng/ml) were significantly higher than those of patients with high serum sIL-27Rα (<59.40 ng/ml). Multivariate analysis confirmed that low sIL-27Rα level (HR = 2.83 95% CI 1.29–6.19, *P* < 0.01) was an independent risk factor for predicting grade II-IV aGVHD. In addition, serum sIL-27Rα was positively correlated with IL-27 (R = 0.27, *P* = 0.029), IL-10 (R = 0.37, *P* = 0.0015) and HGF (R = 0.27, *P* = 0.0208), but was negatively correlated with TNFR1 (R = −0.365, *P* = 0.0022) and ST2 (R = −0.334, *P* = 0.0041), elafin (R = −0.29, *P* = 0.0117), and REG3α (R = −0.417, *P* = 0.0003). More importantly, the threshold value of sIL-27Rα was then validated in an independent cohort of 80 patients (AUC = 0.790, 95% CI 0.688–0.892, *P* < 0.001). Taken together, our findings suggested that serum sIL-27Rα at neutrophil engraftment maybe a valuable prognostic biomarker in predicting the incidence of moderate-to-severe aGVHD.

## Introduction

Allogeneic haematopoietic stem cell transplantation (allo-HSCT) is currently one of the effective means to cure a variety of malignant and nonmalignant haematological diseases^1^. Acute graft versus host disease (aGVHD), one of the leading causes of transplant-related death, is a major and serious complication after allo-HSCT, with an incidence of approximately 40–60%^2,3^. Despite greater understanding of the pathogenesis of aGVHD, the diagnosis of aGVHD relies primarily on clinical symptoms of aGVHD target organs, and its incidence and mortality remains very high^4^.

Plasma biomarkers have emerged as important tools for the diagnosis of aGVHD. Examples include elafin for skin GVHD^5^, regenerating islet-derived 3α (REG3α) for gastrointestinal GVHD^6^, hepatocyte growth factor (HGF), cytokeratin fragment 18 (KRT18) for liver GVHD^7,8^, interleukin-2 receptor α (IL-2Rα), tumour necrosis factor receptor 1 (TNFR1), IL-8, IL-7, and soluble DNAX accessory molecule-1 (DNAM-1) for systemic GVHD^7,9,10^. In addition, serum stimulation-2 (ST2) and the plasma microRNA signature can serve as prognostic indicators of treatment-resistant GVHD^11,12^.

IL-27 is a heterodimeric cytokine of the IL-12 family, composed of two subunits, EBI3 and p28^13^. IL-27 signals through a heterodimeric receptor composed of gp130 and IL-27Rα^14^, leading to activation of the STAT pathway. IL-27Rα (formerly called WSX1 or TCCR) was first cloned in 1998 and is highly expressed on effector and memory CD4^+^ and CD8^+^ T cells^15,16^. A previous study by Odile *et al*.^17^ demonstrated that membrane IL-27Rα existed in its soluble form (sIL-27Rα) in healthy human serum as well as in the serum of patients with Crohn’s disease, suggesting that sIL-27Rα may play an essential role in normal as well as pathological conditions. sIL-27Rα can be produced by human activated CD4^+^ and CD8^+^ T cells, B cells, myeloid cells, and various cell lines. More importantly, they found that sIL-27Rα bound IL-27 and antagonized IL-27 signalling, thus functioning as a natural antagonist of IL-27 under normal and pathological conditions^17^. However, expression levels and the clinical significance of serum IL-27Rα in patients after allo-HSCT remain largely unknown. It remains unclear as to whether there any associations between sIL-27Rα levels and the development of aGVHD.

In this study, we investigated the expression of human sIL-27Rα in serum of patients with aGVHD after allo-HSCT. We also examined the relationship between serum levels of sIL-27Rα and the development of aGVHD. Our results indicated that sIL-27Rα was a valuable prognostic biomarker for the development of aGVHD after allo-HSCT.

## Patients and Methods

### Patients and samples

A total of 72 patients with full clinical follow-up data who underwent allo-HSCT at the First Affiliated Hospital of Soochow University were recruited in our Biobank from Jan 1, 2012 to Dec 31, 2012 and were selected as the training group. Another 80 cases with full clinical follow-up data were recruited in our Biobank from Jan 1, 2013 to Dec 31, 2013 and were selected as the validation group. The characteristics of 152 total cases are shown in Table S1. The characteristics of the patients in the training and validation groups were similar. The bone marrow human leucocyte antigens (HLA-A, HLA-B, HLA-C, HLA-DRB1, HLA-DQB1) in these patients were obtained by high-resolution deoxyribonucleic acid techniques^18^, including compatriots in full, kinship not all in full and unrelated in full. All patients were pretreated with a myeloablative conditioning regimen. Myeloablative conditioning regimens contained high-dose cyclophosphamide (CTX) with busulfan. The day of donor cell infusion was day 0. Recipients were given immunosuppressive drugs, including a calcineurin inhibitor and methotrexate to prevent aGVHD. The diagnosis of aGVHD was primarily based on the clinical and pathological findings of the patients and was graded according to consensus criteria^19^. Serum samples were obtained before initiating the conditioning regimen and on the day of neutrophil engraftment. The date of neutrophil engraftment was defined as the first of three consecutive days when the patient’s neutrophil counts exceeded 0.5 × 10^9^/L, and serum samples were collected at the first day post neutrophil engraftment. This study was approved by the ethics committee of the First Affiliated Hospital of Soochow University. Written informed consent was obtained from each patient in accordance with the Declaration of Helsinki.

### Measuring serum sIL-27Rα levels

Serum levels of sIL-27Rα was measured by sandwich enzyme-linked immunosorbent assay (ELISA) as described previously^17^. Briefly, 96-well plates were coated with mouse anti-human IL-27Rα monoclonal antibody (Clone #191106, R&D Systems, 0.8 μg/ml, 100 μL/well) overnight at room temperature, The plates were blocked with reagent diluent (1% BSA in PBS, 300 μL/well, Catalogue #DY995, R&D Systems) for 1 h at room temperature, and then washed three times using washing buffer (0.05% Tween 20 in PBS, 300 μL/well, Catalogue #WA126, R&D Systems). Recombinant human IL-27Rα-Fc chimaera protein (Catalogue #1479-TC-050, R&D Systems) was used as a standard. A total of 100 μL of serum samples or standards were added in each well and incubated 1 h at room temperature. After washing, biotinylated goat anti-human IL-27Rα polyclonal antibody (100 μL/well, Catalogue #BAF1479, R&D Systems) was added as detection antibody for 2 h at room temperature. The plates were then washed and incubated with streptavidin HRP (1:40 in reagent diluent, 100 μL/well, R&D Systems) for 20 min at room temperature. Following another washing, 100 μL of substrate solution (Catalogue #DY999, R&D Systems) was added into each well and incubated for 20 min at room temperature. The reactions were stopped with 2 N H_2_SO_4_. The optical density of each well was measured at 450 nm using a microplate reader (Synergy HT, BioTek, USA). The limit of sensitivity was 30 pg/ml sIL-27Rα. Serum levels of IL-27, IL-10, TNFR1, ST2, elafin and REG3α were measured using a commercial ELISA kit (R&D Systems). HGF was measured using an ELISA kit from Multisciences (Hangzhou, China). The experiments were approved by the ethics committee of the First Affiliated Hospital of Soochow University, and all experiments were performed in accordance with relevant guidelines and regulations.

### Statistical analysis

The association between serum sIL-27Rα levels on the day of neutrophil engraftment and the acute GVHD grade was evaluated using Spearman’s rank correlation coefficient. Continuous and dichotomous variables in the two groups were compared using the Mann–Whitney U test and Fisher’s exact test, respectively. Receiver operator characteristic (ROC) curves were constructed for sIL-27Rα levels predicting the occurrence of acute GVHD, and the area under the ROC curve was calculated to examine the level’s performance. When obtained the largest Youden Index, the corresponding value was cut-off value. The cut-off value from the ROC curves was evaluated for sensitivity and specificity. The relapse rate (CIR) and non-relapse mortality (NRM) were calculated using Gray’s test, and each event was considered a competing risk^20^. The cumulative incidence of acute GVHD was also calculated using Gray’s test, and death without acute GVHD or relapse was considered a competing risk^20^. Factors that exhibited at least marginal significance (*P* < 0.10) in the univariate analyses were included in the multivariate analyses using Fine and Gray proportional hazards model, and subsequently deleted from the model in an enter manner. Overall survival (OS) was estimated using the Kaplan-Meier method and was compared between the groups using the log-rank test. Factors with *P* values of <0.05 were considered statistically significant.

## Results

### Expression levels of sIL-27Rα in GVHD patients

**Figure 1:**
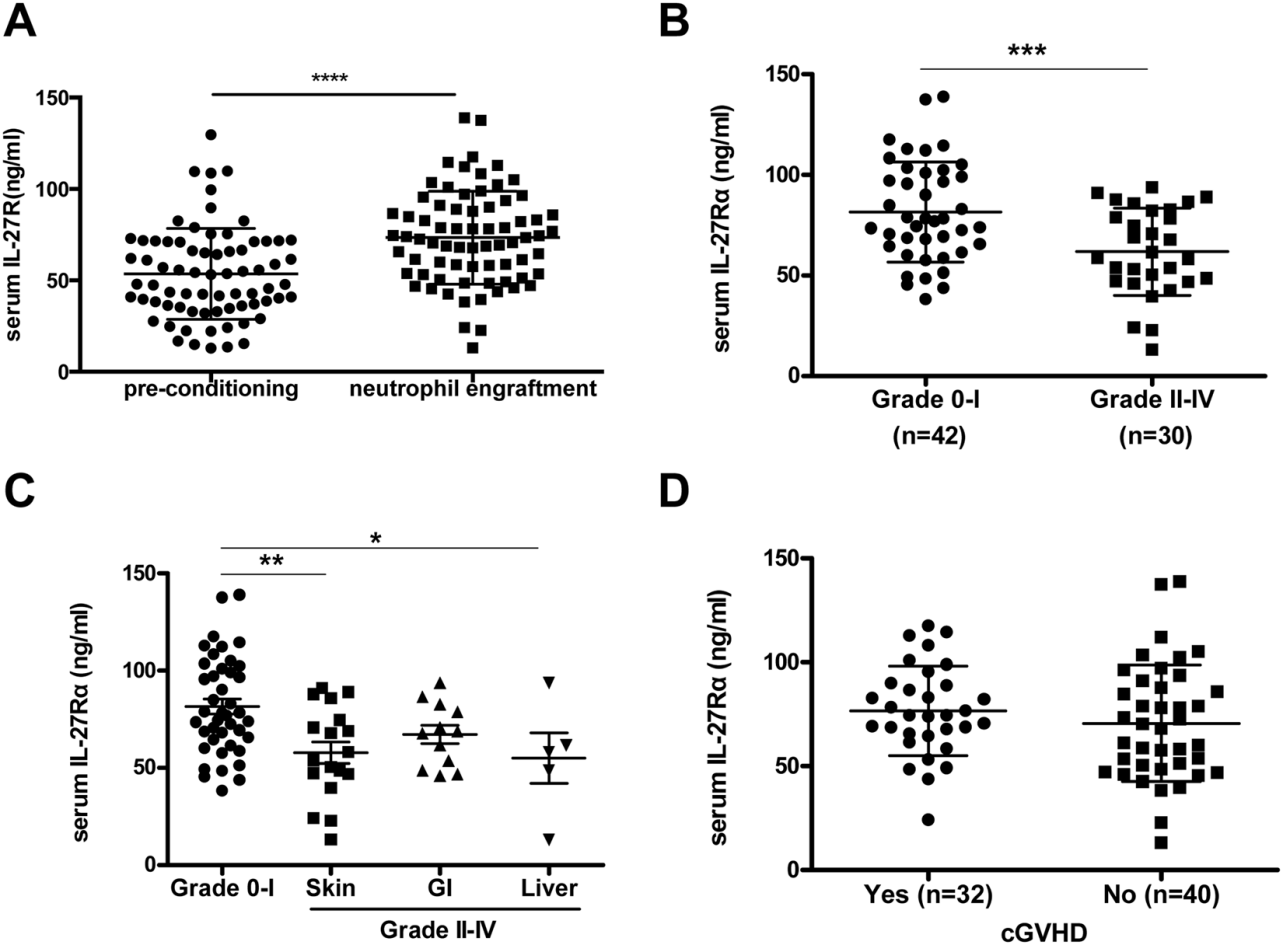
The expression of sIL-27Rα level in GVHD patients. (**A**) Serum sIL-27Rα levels were significantly increased on the day of neutrophil engraftment compared with pre-conditioning (*P* < 0.0001). (**B**) The serum sIL-27Rα levels in patients with grade II–IV aGVHD were significantly lower than those of 0-I aGVHD patients on the day of neutrophil engraftment (*P* < 0.001). (**C**) On the day of neutrophil engraftment, serum sIL-27Rα levels in patients with skin aGVHD (*P* < 0.01) and liver aGVHD (*P* < 0.05) were significantly lower than those of 0-I aGVHD patients. (**D**) There were no similar significant difference regarding cGVHD. Data shown are mean ± SD.

To detect the soluble form of IL-27Rα (sIL-27Rα) in human serum, we developed a sandwich ELISA system and evaluated concentrations of sIL-27Rα in the training set (n = 72). As shown in Fig. 1A, the serum sIL-27Rα levels at the time of pre-conditioning and neutrophil engraftment were 53.52 ± 24.86 ng/ml and 73.40 ± 25.42 ng/ml, respectively; significant upregulation of sIL-27Rα levels was observed after allo-HSCT (Fig. 1A, *P* < 0.001). In addition, serum sIL-27Rα levels in patients with grade II–IV aGVHD were significantly lower than those of 0-I aGVHD patients on the day of neutrophil engraftment (grade 0-I: 81.59 ± 24.93 ng/ml; grade II–IV: 61.92 ± 21.68 ng/ml; *P* < 0.01, Fig. 1B), However, no statistically significant difference in serum sIL-27Rα levels between 0-I aGVHD patients and II–IV aGVHD patients was found at pre-conditioning (Fig. S1A). Moreover, we found that serum sIL-27Rα levels in patients with liver aGVHD (n = 5), but not those with skin aGVHD, and gastrointestinal (GI) aGVHD were significantly lower than those of 0-I aGVHD patients (*P* < 0.05, Fig. S1B) at pre-conditioning. On the day of neutrophil engraftment, serum sIL-27Rα levels in patients with skin aGVHD (n = 18, *P* < 0.01) and liver aGVHD (n = 5, *P* < 0.05) were significantly lower than those of 0-I aGVHD patients (Fig. 1C). However, no statistically significant difference in the serum sIL-27Rα levels between cGVHD (n = 32) and non-cGVHD patients (n = 40) were observed at the two time points (Figs 1D and S1C). Taken together, our results suggest that the sIL-27Rα in the serum as associated with the development of aGVHD.

### The prognostic value of sIL-27Rα in aGVHD

**Figure 2:**
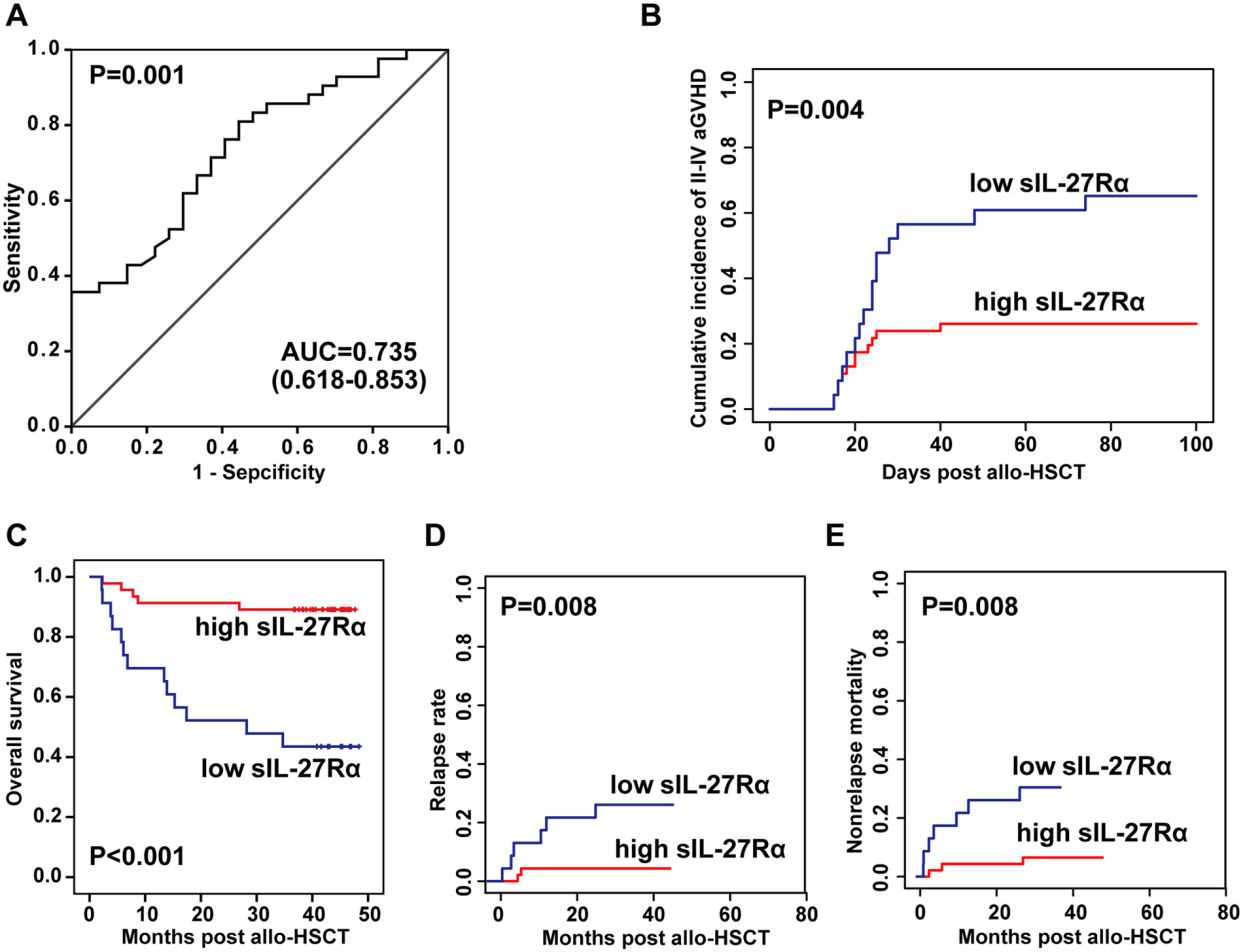
The diagnostic value of sIL-27Rα in aGVHD and the association of sIL-27Rα with aGVHD severity, relapse and survival. (**A**) The area under the ROC curve (AUC) was 0.735 (95% CI 0.618–0.853, *P* = 0.001) on the day of neutrophil engraftment; when using 59.4 ng/ml as cut-off value from the ROC curve, the sensitivity and specificity were 56% and 81%, respectively. (**B**) The cumulative incidence of grade II–IV aGVHD was significantly lower in patients with high sIL-27Rα levels by Gray’s test (*P* = 0.004). (**C**) Patients with high sIL-27Rα levels showed favourable overall survival compared with patients with low sIL-27Rα levels with Kaplan-Meier survival analysis by log rank test (*P* < 0.001). (**D**,**E**) Patients with high sIL-27Rα levels had lower relapse rate (CIR) and non-relapse mortality (NRM) than did patients with low sIL-27Rα levels by Gray’s test (P = 0.008, respectively).

We then analysed the prognostic ability of sIL-27Rα during pre-conditioning, and on the day of neutrophil engraftment for occurrence of II-IV aGVHD by conducting ROC. As shown in Figs S1D and Fig. 2A, the area under the ROC curve (AUC) was 0.556 (95% CI 0.419–0.693, *P* = 0.421) during pre-conditioning and was 0.735 (95% CI 0.618–0.853, *P* = 0.001) on the day of neutrophil engraftment, suggesting that the sIL-27Rα levels on the day of neutrophil engraftment could be used to predict grade II–IV aGVHD; when we used 59.4 ng/ml as the cut-off value for the ROC curve, the sensitivity and specificity were 56% and 81%, respectively with the largest Youden Index (Fig. 2A).

### Association of sIL-27Rα with aGVHD severity, relapse and survival

Next, the patients were divided into two groups according to this cut-off value, of which 46 patients had an sIL-27Rα levels ≥59.40 ng/ml and 23 had an sIL-27Rα levels <59.40 ng/ml. Three patients among them were excluded because they developed grade II–IV aGVHD before neutrophil engraftment. The association of sIL-27Rα with clinical factors was first evaluated. As shown in Table 1, donor type, disease risk levels, aGVHD grade, and patients’ outcomes were significantly associated with low serum sIL-27Rα levels. In addition, the cumulative incidence of grade II–IV aGVHD was significantly lower in patients with high sIL-27Rα levels than in patients with low sIL-27Rα levels (*P* = 0.004, Fig. 2B).

**Table 1:** The association of sIL-27Rα levels at neutrophil engraftment with clinical factors.

Factors	Total	sIL-27Rα ≥ 59.40	sIL-27Rα < 59.40	*P* value
Age median	28(3–59)	29(9–59)	27(3–50)	0.113
Gender				
male	42	27	15	0.601
female	27	19	8	
Donor age	34(16–52)	34(16–52)	36(21–52)	0.508
Patient-donor sex match				
match	44	29	15	0.859
mismatch	25	17	8	
GVHD				
CsA based	49	35	14	0.189
Fk506 based	20	11	9	
Donor type				
related	48	36	12	0.026
unrelated	21	10	11	
Diagnosis				
AML	28	17	11	0.114
ALL	25	15	10	
MDS	6	4	2	
CML	10	10	0	
Disease status				
standand risk	47	35	12	0.045
high risk	22	11	11	
aGVHD grade				
0	14	10	4	0.027
I	28	24	4	
II	17	7	10	
III	6	3	3	
IV	4	2	2	
II–IV aGVHD ggvgvaGVHD organ				
skin	16	6	10	0.975
colon	12	5	7	
liver	5	2	3	
Prognosis				
survival	51	41	10	<0.001
relapse	8	2	6	
other	10	3	7	

Note: 3 patients were excluded because they developed grade II–IV aGVHD before neutrophil engraftment.

The median follow-up of the patients was 41.7 months (range 2.2–48.4) after allo-HSCT. Kaplan-Meier survival analysis showed that patients with low sIL-27Rα levels showed poor overall survival compared with patients with high sIL-27Rα levels (log rank test, *P* < 0.001, Fig. 2C). Moreover, patients with low sIL-27Rα levels had higher relapse rate (CIR) and non-relapse mortality (NRM) than did patients with high sIL-27Rα levels (*P* = 0.008, respectively; Fig. 2D,E). Univariate analyses showed that sIL-27Rα levels <59.40 ng/ml (*P* = 0.001), donor type (*P* = 0.011) and disease status (*P* = 0.016) were significantly associated with poor overall survival (Fig. S2A). Cox survival hazards model analysis confirmed that low sIL-27Rα level (HR = 4.143, 95% CI 1.361–12.614, *P* = 0.012) was the parameter most strongly associated with poor overall survival (Fig. S2B).

### sIL-27Rα as an independent prognostic aGVHD biomarker

To investigate the specificity of sIL-27Rα as a predictor of aGVHD, we performed univariate analyses. The results showed that sIL-27Rα levels <59.40 ng/ml (*P* < 0.01), donor age (*P* = 0.04) and disease status (*P* = 0.08) at neutrophil engraftment were significantly associated with grade II–IV aGVHD (Table 2). Fine and Gray proportional hazards model analysis confirmed that low sIL-27Rα level (HR = 2.83, 95% CI 1.29–6.19, *P* < 0.01) was the parameter most strongly associated with II–IV aGVHD (Table 2), suggesting that low sIL-27Rα level may be an independent risk factor for predicting grade II–IV aGVHD.

**Table 2:** Univariate and multivariate analyses of factors affecting the incidence of grade II–IV acute graft- versus-host disease after allogeneic hematopoietic stem cell transplantation at neutrophil engraftment.

Factor	Univariate analysis		Multivariate analysis		
	Incidence of acute GVHD (%)	P value	Hazard ratio	95% CI	P value
Patient age (years)					
<50	39.1	0.94			
≥50	40.0				
Gender					
Male	40.0	0.11			
Female	51.9				
GVHD prophylaxis					
FK506	45.0	0.57			
CSA	36.7				
Donor age					
<35	27.8	0.04	1	1.11–4.82	0.03
≥35	51.5		2.31		
sIL-27Rα level					
<59.40	65.2	<0.01	2.83	1.29–6.19	<0.01
≥59.40	26.1		1		
Disease status					
High risk	54.5	0.08	1.42	0.69–2.97	0.34
Standard risk	31.9		1		
Patient-donor sex					
match	38.6	0.81			
mismatch	40.0				
Donor type					
Unrelated	42.9	0.98			
Related	37.5				

Note: 3 patients were excluded because they developed grade II–IV aGVHD before neutrophil engraftment.

**Figure 3:**
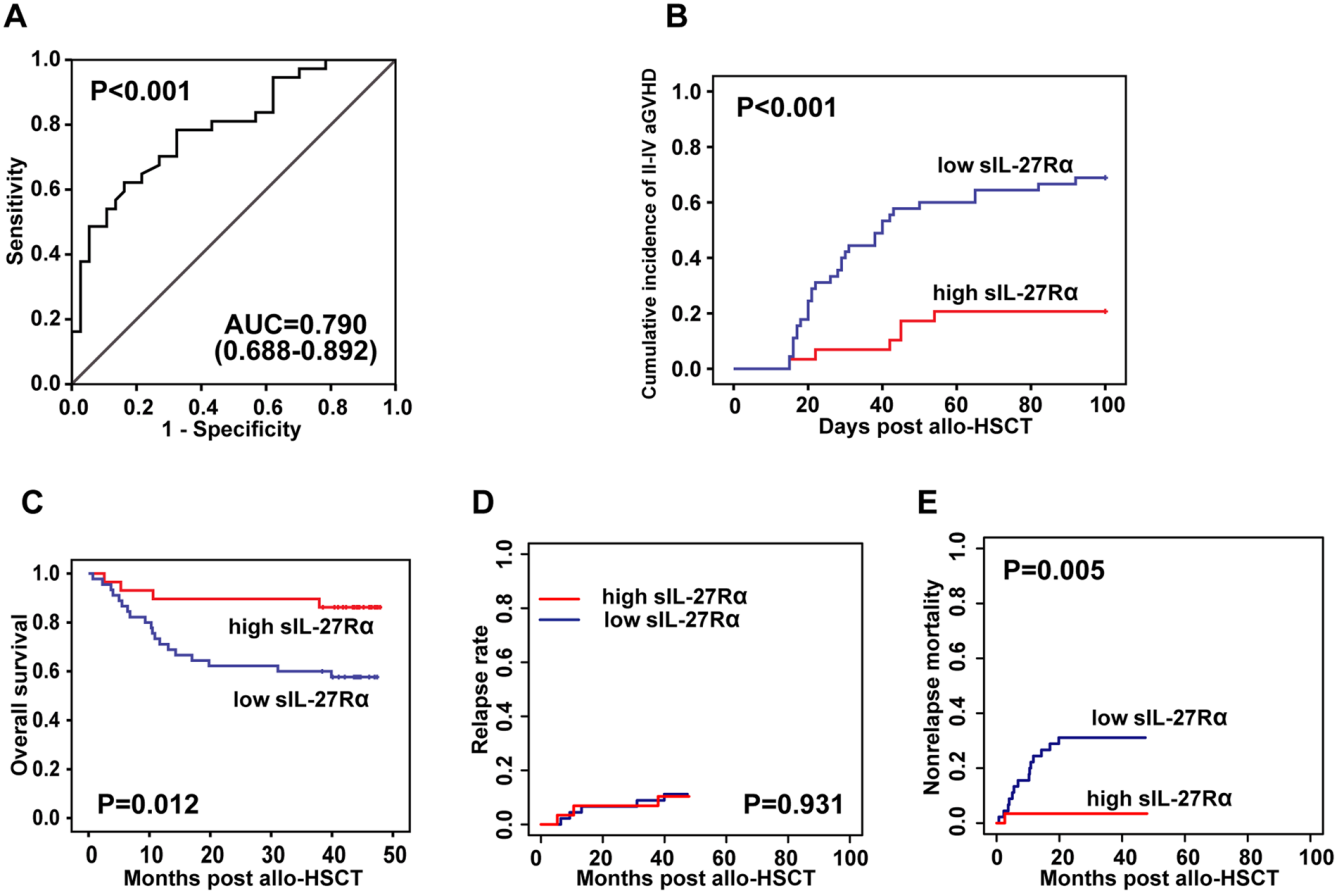
sIL-27Rα as an independent prognostic aGVHD biomarker validated in an independent cohort of 80 patients. (**A**) The area under the ROC curve (AUC) was 0.790 (95% CI 0.688–0.892, *P* < 0.001) on the day of neutrophil engraftment, using 59.4 ng/ml as the cut-off value. (**B**) The cumulative incidence of grade II–IV aGVHD was significantly lower in patients with high sIL-27Rα levels by Gray’s test (*P* < 0.001). (**C**) Patients with high sIL-27Rα levels showed favourable overall survival compared with patients with low sIL-27Rα levels on Kaplan-Meier survival analysis by log rank test (*P = *0.012). (**D**,**E**) Patients with high sIL-27Rα levels had similar relapse rates (CIR) and lower non-relapse mortality (NRM) than did patients with low sIL-27Rα levels by Gray’s test (*P* = 0.0931, *P* = 0.005, respectively).

The prognostic value of sIL-27Rα was validated in a second independent cohort of 80 patients (Table S1). In the validation group, 40 patients developed grade II–IV acute GVHD, 6 of whom were excluded because they developed grade II–IV aGVHD before neutrophil engraftment. The same threshold of sIL-27Rα (59.4 ng/ml) was applied in the independent validation set. As shown in Fig. 3A, the AUC was 0.790 (95% CI 0.688–0.892, *P* < 0.001), with sensitivity and specificity 83.8% and 62.2%, respectively. In addition, patients with low sIL-27Rα levels showed a high cumulative incidence of grade II–IV aGVHD (*P* < 0.001, Fig. 3B), poor overall survival (*P* < 0.05, Fig. 3C), and higher non-relapse mortality (*P* = 0.005, Fig. 3E), than did patients with high sIL-27Rα levels. However, there were no significant differences in relapse rates between the two groups (*P* = 0.931, Fig. 3D). Taken together, our results suggested that sIL-27Rα in serum was a valuable prognostic biomarker for II–IV aGVHD after allo-HSCT.

### Association of sIL-27Rα with serum cytokine levels in aGVHD

**Figure 4:**
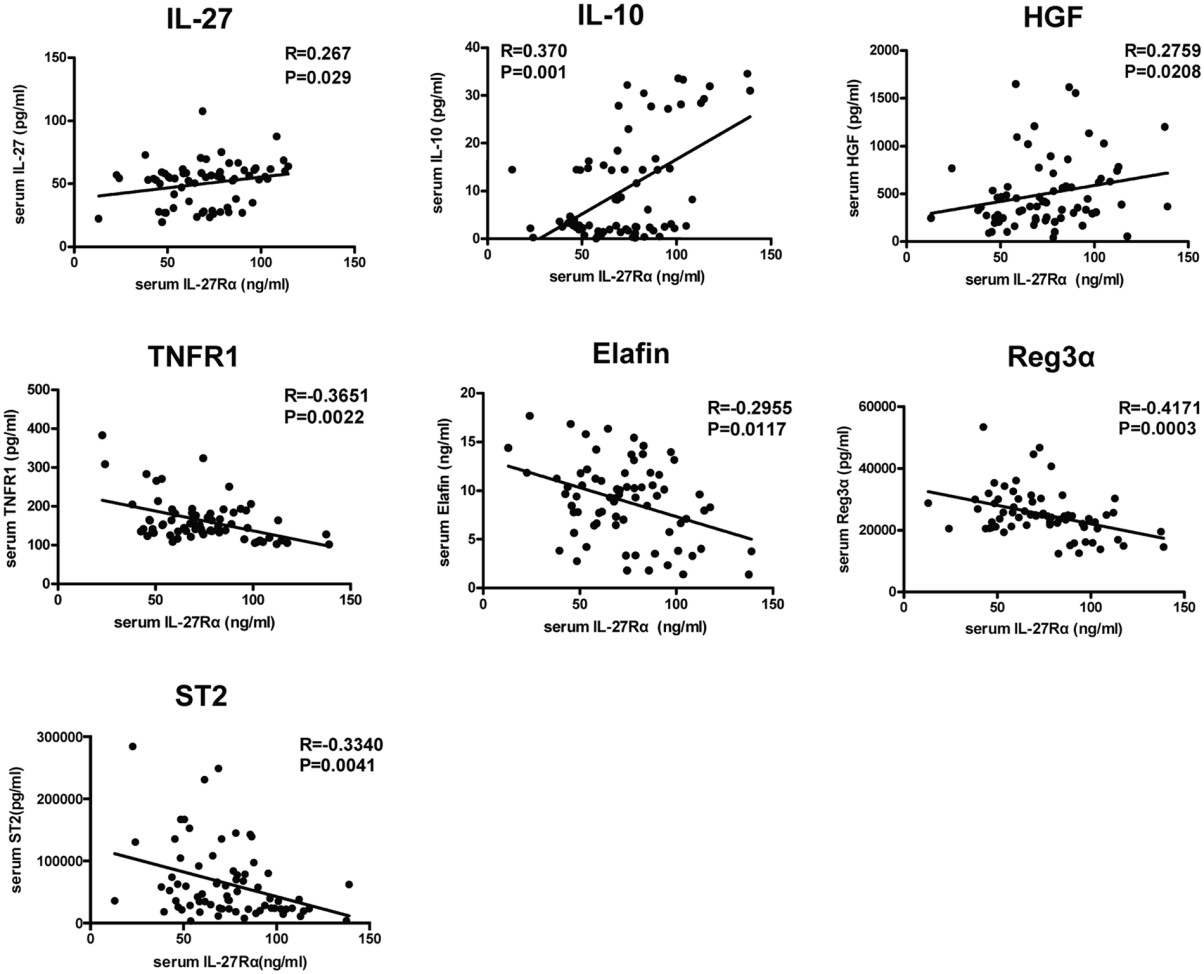
Association of sIL-27Rα with serum cytokine levels in aGVHD. The associations between sIL-27Rα levels with serum IL-27, IL-10, HGF, TNFR1, elafin, REG3α and ST2 levels were analysed by using Spearman’s rank correlation coefficient.

The above-referenced data demonstrated that low serum sIL-27Rα levels at neutrophil engraftment were associated with II-IV aGVHD, poor OS and high relapse rate, suggested that sIL-27Rα may play a protective role in aGVHD development. To investigate the potential mechanism, we examined the expression of inflammatory cytokines at neutrophil engraftment, as well as certain identified plasma aGVHD biomarkers and evaluated their correlations with sIL-27Rα levels. As shown in Fig. 4, sIL-27Rα levels were positively correlated with IL-27 (R = 0.27, *P* = 0.029), anti-inflammatory cytokine IL-10 (R = 0.37, *P* = 0.0015) and liver GVHD biomarker HGF (R = 0.27, *P* = 0.0208), but were negatively correlated with TNFR1 (R = −0.365, *P* = 0.0022) and ST2 (R = −0.334, *P* = 0.0041), skin GVHD biomarker elafin (R = −0.29, *P* = 0.0117), and gastrointestinal GVHD biomarker REG3α (R = −0.417, *P* = 0.0003).

Additionally, it has previously been shown that absolute lymphocyte counts were correlated with incidence of GVHD^21,22^. However, our data showed that there was no significant difference in absolute lymphocyte counts between grade 0-I aGVHD and grade II–IV aGVHD (*P* = 0.859, data not shown), and no significant correlation between absolute lymphocyte counts and serum sIL-27Rα levels (R = −0.149, *P* = 0.93, data not shown). Furthermore, we also assessed the above 7 biomarkers on the day of neutrophil engraftment and evaluated their prognostic value in grade II–IV aGVHD. The results showed that TNFR1 (AUC = 0.664, 95% CI 0.536–0.793, *P* = 0.022), ST2 (AUC = 0.724, 95% CI 0.603–0.845, *P* = 0.002), IL-27 (AUC = 0.645, 95% CI 0.510–0.780, *P* = 0.043) may be used to predict grade II–IV aGVHD (Table S3). Multivariate analysis showed that sIL-27Rα and ST2 were the best biomarkers to predict II–IV aGVHD after allo-HSCT (Table S4).

## Discussion

aGVHD symptoms are the result of damaged target tissues with the release of a large number of cytokines and proteins at the onset of aGVHD^3^. Therefore, plasma proteomic profiles could be used as biomarkers for diagnosis of aGVHD and prediction of prognosis of aGVHD. In this study, we identified for the first time that serum sIL-27Rα = 59.40 ng/ml at neutrophil engraftment after allo-HSCT was the threshold for predicting the development of II–IV aGVHD. However, since the AUC value of sIL-27Rα determined by ROC curves may be less efficient, a single sIL-27Rα marker at neutrophil engraftment may not be enough for accurate prediction of aGVHD, and combination with other biomarkers, if any, could predict aGVHD occurrence after transplantation.

IL-27 is a potent inflammatory cytokine with immune regulatory properties. It was first identified as a pro-inflammatory cytokine that induced proliferation of CD4^+^ T cells and production of IFN-γ^23,24^. However, subsequent studies revealed that IL-27 had inhibitory effects by inducing IL-10 producing Treg cells and Tr1 cells^25,26^. IL-27 are mainly produced by activated antigen-presenting cells, including dendritic cells and macrophages, and its receptor, IL-27Rα and gp130, are constitutively expressed by numerous immune on nonimmune cells^14^. Some cytokine receptors, including ST2, gp130, IL-15Rα, and IL-7Rα can exist in both membrane and soluble forms^27–30^. Their soluble form can act as an agonist or antagonist by binding to its ligand. sIL-27Rα was first reported in mouse neuronal cells as an alternatively spliced isoform missing exons 7–14^31^. This truncated IL-27Rα isoform acted as a functional subunit of a receptor for an anti-Alzheimer’s disease rescue factor Humanin^31^. Later, the Odile group first reported that IL-27Rα existed naturally as a soluble form that antagonized IL-27 activity in activated T cell culture supernatants, healthy human serum, and Crohn’s disease patients^17^, suggesting that sIL-27Rα may play an essential role in normal and pathological conditions. However, the clinical significance of serum IL-27Rα levels remained largely unknown.

In this study, using an ELISA method described previously^17^, we detected the concentrations of sIL-27Rα in the sera of aGVHD and non-GVHD patients before initiating the conditioning regimen and on the day of neutrophil engraftment. Our results showed that only sIL-27Rα levels at neutrophil engraftment could predict grade II–IV aGVHD development. The cumulative incidence of grade II–IV and NRM was similar between high and low sIL-27Rα at pre-conditioning (Fig. S3A,D), while low sIL-27Rα levels showed slightly poor overall survival, and lower CIR compared to that of patients with high sIL-27Rα levels at pre-conditioning (Fig. S3B,C). Univariate and multivariate analyses demonstrated that low sIL-27Rα levels at pre-conditioning were not significantly associated with grade II–IV aGVHD (Table S2) and overall survival (Fig. S4A,B). The prognostic value of sIL-27Rα was further validated in a second independent cohort of 80 patients. Therefore, our results indicated that sIL-27Rα levels at neutrophil engraftment after allo-HSCT may be a useful predictor of II–IV aGVHD.

In this study, we found that sIL-27Rα was significantly down-regulated in patients with grade II–IV aGVHD, especially in patients with skin or liver aGVHD. Patients with low sIL-27Rα levels showed higher incidences of grade II–IV aGVHD, poor overall survival, and higher recurrence rates than did patients with high sIL-27Rα levels, suggesting that sIL-27Rα may play a protective role in the development of aGVHD. We also give some clues regarding its mechanism. We found that sIL-27Rα was positively associated anti-inflammatory cytokine IL-10 and HGF, while negatively associated with TNFR1, elafin and REG3α. IL-10 and HGF have been shown to ameliorate aGVHD^32,33^, whereas TNFR1, elafin, REG3α were associated with severe systemic, skin and gastrointestinal aGVHD^5,7^. Previous studies showed that IL-27 played a critical role in the parent-to-F1 model of aGVHD^34^. A subsequent study using IL-27-deficient mice showed that the role of IL-27 in aGVHD was controversial. Recipient mice reconstituted with marrow grafts from IL-27 p28-deficient donors exacerbated aGVHD, while those treated with p28 antibody had significantly reduced aGVHD^35^, indicating that IL-27 signalling had a complicated role in the development of aGVHD. In our aGVHD samples, we also detected high levels of IL-27 in serum of aGVHD patients, and patients with high IL-27 levels showed lower incidences of grade II–IV aGVHD as well as favourable overall survival (data not shown), suggesting that IL-27 played a protective role in the development of aGVHD. We also found that sIL-27Rα levels were positively correlated with IL-27 levels (R = 0.27, *P* = 0.029), in accordance with similar observations in Crohn’s disease patients, suggesting that the interaction between IL-27 and sIL-27Rα may play critical role in aGVHD biology. However, the underling mechanisms remain under further investigation.

In conclusion, we measured sIL-27Rα in the serum of allo-HSCT patients and demonstrated that sIL-27Rα can potentially be a useful biomarker for prediction of the development of aGVHD. A multicentre, prospective study is needed to validate our findings. Further study is required to clarify the pathological functions of sIL-27Rα in the development of aGVHD.

### Ethics Statement

This study was approved by the ethics committee of the First Affiliated Hospital of Soochow University. Written informed consent was obtained from each patient in accordance with the Declaration of Helsinki.

## Electronic supplementary material

Supplementary Information
