# Supplementary material for: Soluble interleukin-27 receptor alpha is a valuable prognostic biomarker for acute graft-versus-host disease after allogeneic haematopoietic stem cell transplantation
Source: Sci Rep. 2018 Jul 9;8:10328. doi: 10.1038/s41598-018-28614-4 (PMC6037712; doi:10.1038/s41598-018-28614-4)
Supplement: Supplementary file 1 — Supplementary Information [file 41598_2018_28614_MOESM1_ESM.docx]

**Soluble interleukin-27 receptor alpha is a valuable prognostic biomarker for acute graft-versus-host disease after allogeneic haematopoietic stem cell transplantation**

Shuangzhu Liu ^1,3,5^, Jingjing Han^1,3,5^, Huanle Gong^2,5^, Yongsheng Li^4,5^, Xiebing Bao^1^, Jiaqian Qi^1^, Hong Liu^1,3^, Jia Chen^1,3^, Xiaojin Wu^1,3^, Yang Xu^1,2,3^, Shoubao Ma^1,2,3*^, Depei Wu^1,2,3*^

^1^Jiangsu Institute of Hematology, The First Affiliated Hospital of Soochow University, Suzhou 215006 China.

^2^Institute of Blood and Marrow Transplantation, Soochow University, Suzhou, 215123 China.

^3^Collaborative Innovation Center of Hematology, Soochow University, Suzhou 215006, China.

^4^Department of Rheumatology, Huai’an First People’s Hospital, Nanjing Medical University, Huai’an 223300 China.

^5^These authors contributed equally to this work.

**Correspondence:** Prof. Depei Wu, Institute of Blood and Marrow Transplantation, Department of Hematology, Collaborative Innovation Center of Hematology, The First Affiliated Hospital of Soochow University, Suzhou 215006, Jiangsu, China. E-mail: [wudepei@medmail.com.cn](mailto:wudepei@medmail.com.cn). Or Dr. Shoubao Ma, Institute of Blood and Marrow Transplantation, Soochow University, Suzhou, 215123 China. E-mail: [mashoubao@suda.edu.cn](mailto:mashoubao@suda.edu.cn)

**Figure S1. The expression of sIL-27Rα level in GVHD patients at pre-conditioning.** (A-B) There were no substantial differences between grade II-IV aGVHD and 0-I aGVHD patients with respect to serum sIL-27Rα levels, while serum sIL-27Rα levels in patients with liver aGVHD (*P*<0.05) were significantly lower than those of 0-I aGVHD patients by Mann-Whitney U test. (C) There were no differences regarding chronic GVHD. (D) The area under the ROC curve (AUC) was 0.556 (95% CI 0.419-0.693, *P*=0.421) at pre-conditioning.


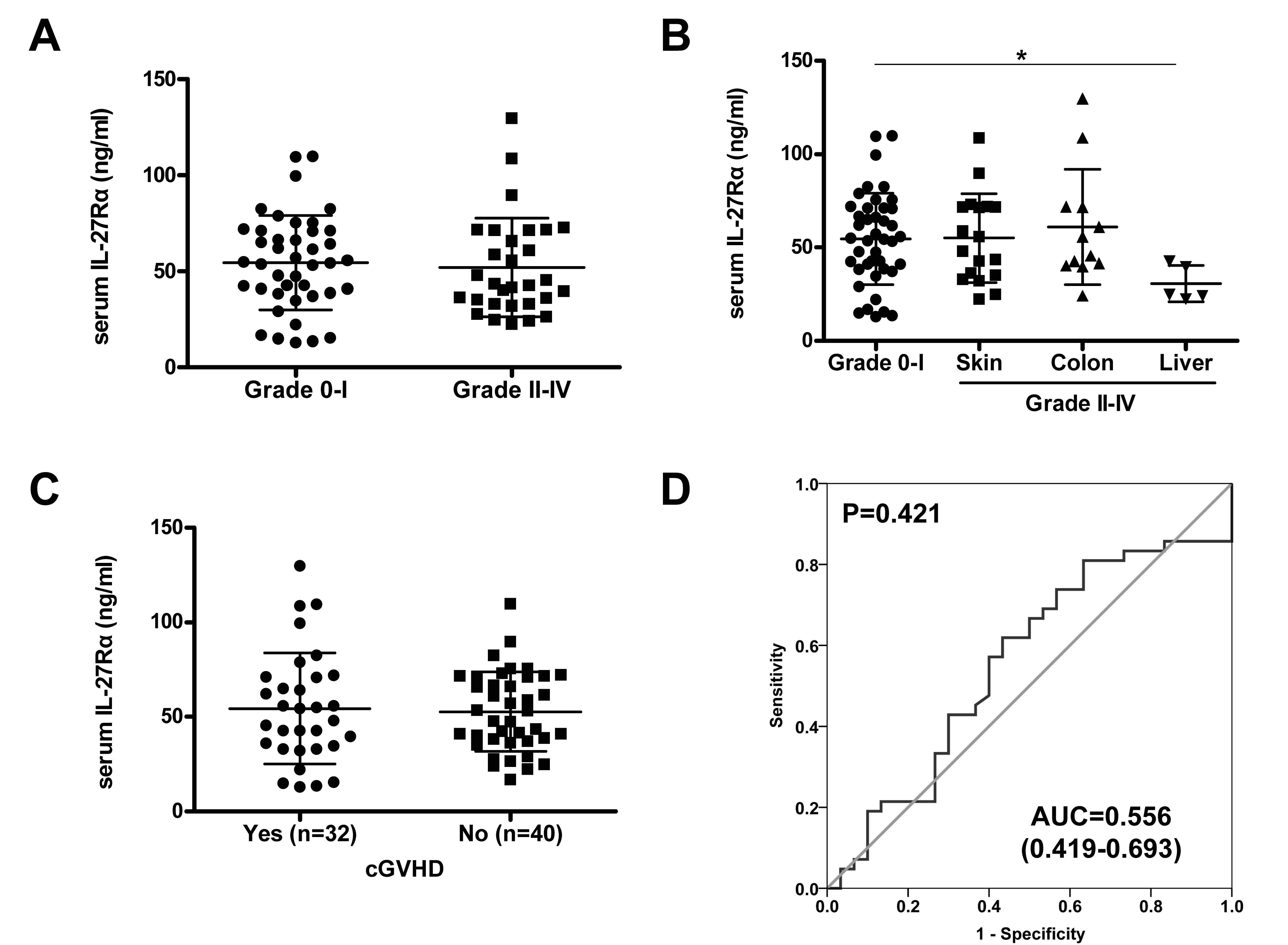


**Figure S2. Univariate and multivariate analyses of factors affecting overall survival after allo-HSCT.** (A) Univariate analyses showed that sIL-27Rα levels <59.40 ng/ml, donor type and disease status were significantly associated with poor overall survival. (B) Cox survival hazards model analysis confirmed that low sIL-27Rα level was the parameter most strongly associated with poor overall survival.


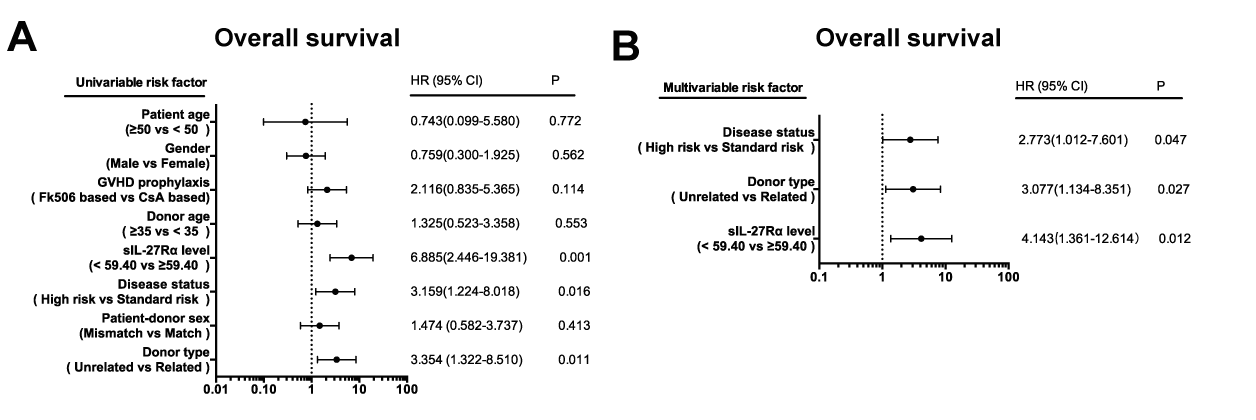


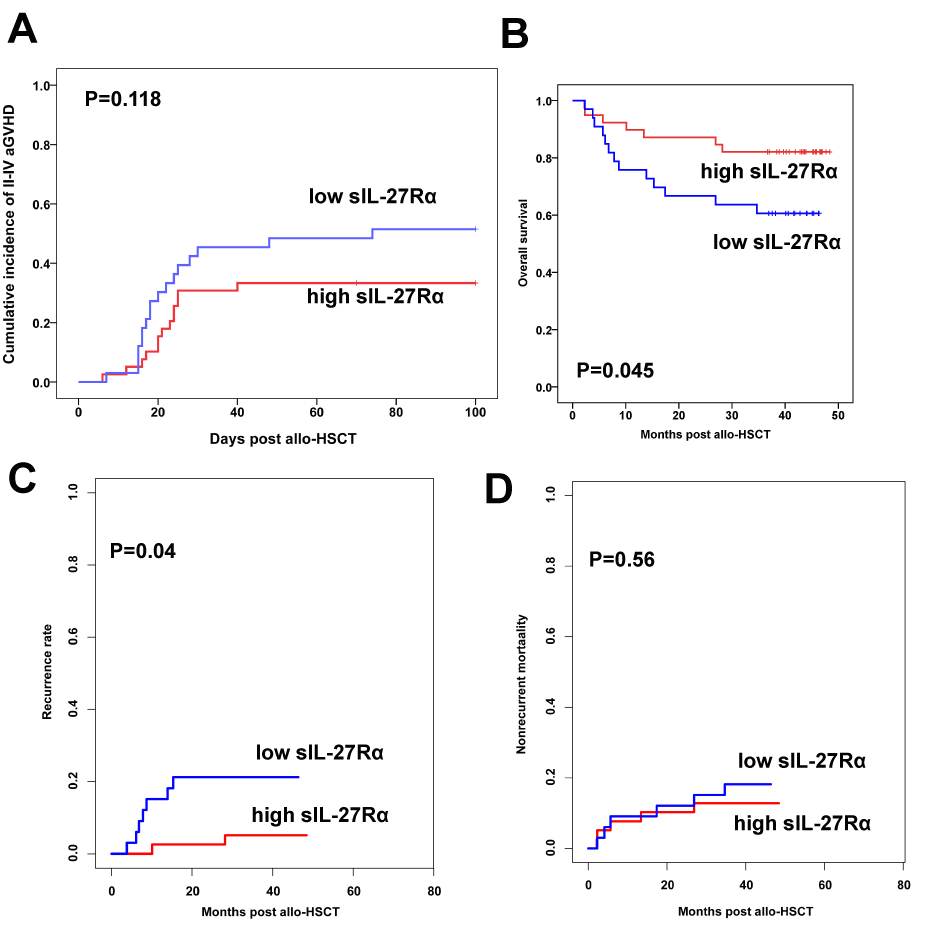
**Figure S3. Association of sIL-27Rα with aGVHD severity, relapse and survival at pre-conditioning.** (A) There were no significant differences regarding cumulative incidence of grade II-IV aGVHD by Gray’s test. (B) Patients with high sIL-27Rα levels showed favourable overall survival compared with patients with low sIL-27Rα levels on Kaplan-Meier survival analysis by log rank test (*P=*0.045). (C) In the case of relapse rate (CIR), patients with high sIL-27Rα levels had lower relapse rates (CIR) than did patients with low sIL-27Rα levels by Gray’s test (*P*=0.04). (D) There were no similar trends for non-relapse mortality (NRM) by Gray’s test.

**Figure S4.** **Univariate and multivariate analyses of factors affecting overall survival at pre-conditioning.** (A) Univariate analyses showed that sIL-27Rα levels, disease status and donor type were associated with overall survival. (B) Multivariate analysis showed that disease status and donor type were the parameters most strongly associated with overall survival.


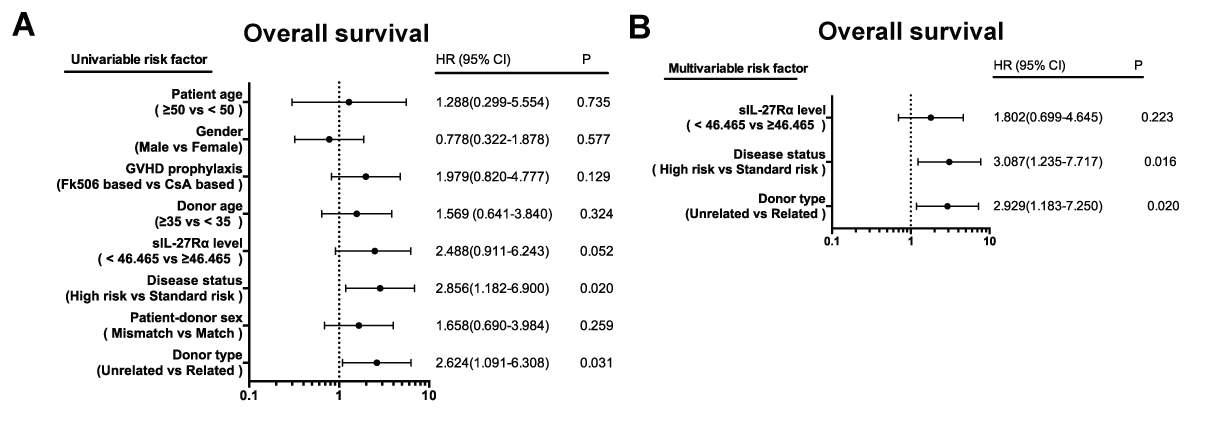


Table S1. Patients and Transplantation Characteristics.

| Factors | Total | Training group | Validation group | P值 |  |
| --- | --- | --- | --- | --- | --- |
|  | (n=152) | （2012, n=69+3 patients developed grade II-IV aGVHD before neutrophil engraftment.） | (2013, n=74+6 patients developed grade II-IV aGVHD before neutrophil engraftment.) |  |  |
| Age Median | 30(3-59) | 28(3-59) | 31(6-52) | 0.802 |  |
| Sex |  |  |  |  |  |
| Male | 94 | 43 | 51 | 0.610 |  |
| Female | 58 | 29 | 29 |  |  |
| Donor age | 34(16-64) | 34(16-55) | 35(20-64) | 0.842 |  |
| Patient-donor sex |  |  |  |  |  |
| match | 90 | 44 | 46 | 0.651 |  |
| mismatch | 62 | 28 | 34 |  |  |
| GVHD prophylaxis |  |  |  |  |  |
| CsA based | 109 | 50 | 59 | 0.556 |  |
| Fk506 based | 43 | 22 | 21 |  |  |
| Donor |  |  |  |  |  |
| related | 102 | 50 | 52 | 0.560 |  |
| Unlated | 50 | 22 | 28 |  |  |
| Diagnosis |  |  |  |  |  |
| AML | 65 | 30 | 35 | 0.329 |  |
| ALL | 50 | 25 | 25 |  |  |
| MDS | 19 | 6 | 13 |  |  |
| CML | 18 | 11 | 7 |  |  |
| Disease status |  |  |  |  |  |
| standand risk | 104 | 48 | 56 | 0.659 |  |
| High risk | 48 | 24 | 24 |  |  |
| aGVHD grade |  |  |  |  |  |
| 0 | 31 | 14 | 17 | 0.675 |  |
| I | 51 | 28 | 23 |  |  |
| II | 45 | 20 | 25 |  |  |
| III | 13 | 6 | 7 |  |  |
| IV | 12 | 4 | 8 |  |  |
| II–IV aGVHD |  |  |  |  |  |
| organ |  |  |  |  |  |
| skin | 43 | 18 | 25 | 0.340 |  |
| colon | 37 | 12 | 25 |  |  |
| liver | 21 | 5 | 16 |  |  |
| Prognosis |  |  |  |  |  |
| survival | 107 | 52 | 55 | 0.744 |  |
| relapse | 18 | 9 | 9 |  |  |
| other | 27 | 11 | 16 |  |  |

Table S2. Univariate and multivariate analyses of factors affecting the incidence of grade II–IV acute graft- versus-host disease after allogeneic hematopoietic stem cell transplantation on at pre-conditioning

| Factor | Univariate analysis | |  | Multivariate analysis | | |
| --- | --- | --- | --- | --- | --- | --- |
|  | Incidence of acute GVHD (%) | P value |  | Hazard ratio | 95% CI | P value |
| Patient age (years) |  |  |  |  |  |  |
| <50 | 40.9 | 0.55 |  |  |  |  |
| ≥50 | 50.0 |  |  |  |  |  |
| Gender |  |  |  |  |  |  |
| Male | 32.6 | 0.06 |  | 1 | 1.19-5.38 | 0.02 |
| Female | 55.2 |  |  | 2.50 |  |  |
| GVHD prophylaxis |  |  |  |  |  |  |
| FK506 | 50.0 | 0.29 |  |  |  |  |
| CSA | 38.0 |  |  |  |  |  |
| Donor age |  |  |  |  |  |  |
| <35 | 29.7 | 0.03 |  | 1 | 0.91-4.15 | 0.08 |
| ≥35 | 54.3 |  |  | 1.95 |  |  |
| sIL-27R level |  |  |  |  |  |  |
| <46.465 | 51.5 | 0.12 |  |  |  |  |
| ≥46.465 | 33.3 |  |  |  |  |  |
| Disease status |  |  |  |  |  |  |
| High risk | 58.3 | 0.04 |  | 2.57 | 1.19-5.54 | 0.02 |
| Standard risk | 33.3 |  |  | 1 |  |  |
| Patient-donor sex |  |  |  |  |  |  |
| match | 38.6 | 0.37 |  |  |  |  |
| mismatch | 46.4 |  |  |  |  |  |
| Donor type |  |  |  |  |  |  |
| Unrelated | 45.5 | 0.93 |  |  |  |  |
| Related | 40.0 |  |  |  |  |  |

Table S3. Analysis of 8 analytes in 69 samples collected on the day of neutrophil engraftment after transplantation.

|  | Area under ROC curve, P value OR (95% CI) |
| --- | --- |
|  | grade 2-4 vs grade 0-1 |
| **TNFR1** |  |
| AUC | 0.664 |
| P | 0.022 |
| OR(95% CI) | 0.536-0.793 |
| **IL-10** |  |
| AUC | 0.509 |
| P | 0.897 |
| OR(95% CI) | 0.367-0.651 |
| **ST2** |  |
| AUC | 0.724 |
| P | 0.002 |
| OR(95% CI) | 0.603-0.845 |
| **sIL-27Rα** |  |
| AUC | 0.735 |
| P | 0.001 |
| OR(95% CI) | 0.618-0.853 |
| **Elafin** |  |
| AUC | 0.551 |
| P | 0.476 |
| OR(95% CI) | 0.410-0.692 |
| **Reg-3α** |  |
| AUC | 0.480 |
| P | 0.782 |
| OR(95% CI) | 0.341-0.619 |
| **HGF** |  |
| AUC | 0.415 |
| P | 0.235 |
| OR(95% CI) | 0.273-0.557 |
| **IL-27** |  |
| AUC | 0.645 |
| P | 0.043 |
| OR(95% CI) | 0.510-0.780 |

Note: 3 patients were excluded because they developed grade II-IV aGVHD before neutrophil engraftment.

Table S4. Univariate and multivariate analyses of biomarkers affecting the incidence of grade II–IV acute graft- versus-host disease after allogeneic hematopoietic stem cell transplantation at neutrophil engraftment

| Factor | Univariate analysis | |  | Multivariate analysis | | |
| --- | --- | --- | --- | --- | --- | --- |
|  | Incidence of acute GVHD (%) | P value |  | Hazard ratio | 95% CI | P value |
| **TNFR1（**pg/ml） |  |  |  |  |  |  |
| <148.02 | 23.5 | 0.02 |  | 1 | 1.06-5.60 | 0.04 |
| ≥148.02 | 54.3 |  |  | 2.43 |  |  |
| **IL-10（**pg/ml） |  |  |  |  |  |  |
| <20.00 | 29.6 | 0.15 |  |  |  |  |
| ≥20.00 | 45.2 |  |  |  |  |  |
| **ST2（**pg/ml） |  |  |  |  |  |  |
| <14133.95 | 21.6 | <0.01 |  | 1 | 1.27-6.89 | 0.01 |
| ≥14133.95 | 59.4 |  |  | 2.96 |  |  |
| **sIL-27Rα（**ng/ml） |  |  |  |  |  |  |
| <59.40 | 65.2 | <0.01 |  | 2.83 | 1.29-6.19 | <0.01 |
| ≥59.40 | 26.1 |  |  | 1 |  |  |
| **Elafin（**pg/ml） |  |  |  |  |  |  |
| <10530.97 | 32.6 | 0.11 |  |  |  |  |
| ≥10530.97 | 52.2 |  |  |  |  |  |
| **Reg-3α（**pg/ml） |  |  |  |  |  |  |
| <23930.40 | 33.3 | 0.36 |  |  |  |  |
| ≥23930.40 | 43.6 |  |  |  |  |  |
| **HGF（**pg/ml） |  |  |  |  |  |  |
| <1585.13 | 36.9 | 0.07 |  |  |  |  |
| ≥1585.13 | 75.0 |  |  |  |  |  |
| **IL-27（**pg/ml） |  |  |  |  |  |  |
| <43.76 | 60.0 | 0.02 |  | 2.36 | 1.10-5.10 | 0.03 |
| ≥43.76 | 27.3 |  |  | 1 |  |  |

Note: 3 patients were excluded because they developed grade II-IV aGVHD before neutrophil engraftment.
